# Melanocortin receptor activation alleviates amyloid pathology and glial reactivity in an Alzheimer’s disease transgenic mouse model

**DOI:** 10.1038/s41598-021-83932-4

**Published:** 2021-02-23

**Authors:** Jackie K. Y. Lau, Min Tian, Yang Shen, Shun-Fat Lau, Wing-Yu Fu, Amy K. Y. Fu, Nancy Y. Ip

**Affiliations:** 1 Division of Life Science, State Key Laboratory of Molecular Neuroscience and Molecular Neuroscience Center, The Hong Kong University of Science and Technology, Clear Water Bay, Hong Kong, China; 2 Hong Kong Center for Neurodegenerative Diseases, Hong Kong, China; 3 Guangdong Provincial Key Laboratory of Brain Science, Disease and Drug Development, HKUST Shenzhen Research Institute, Shenzhen-Hong Kong Institute of Brain Science, Shenzhen, 518057 Guangdong China

**Keywords:** Alzheimer's disease, Neuroimmunology

## Abstract

Alzheimer’s disease (AD) is a devastating neurodegenerative disorder with no disease-modifying treatment. AD progression is characterized by cognitive decline, neuroinflammation, and accumulation of amyloid-beta (Aβ) and neurofibrillary tangles in the brain, leading to neuronal and glial dysfunctions. Neuropeptides govern diverse pathophysiological processes and represent key players in AD pathogenesis, regulating synaptic plasticity, glial cell functions and amyloid pathology. Activation of the pro-opiomelanocortin (POMC)-derived neuropeptide and its receptor from the melanocortin receptor (MCR) family have previously been shown to rescue the impairment in hippocampus-dependent synaptic plasticity in the APP/PS1 mouse model of AD. However, the functional roles of MCR signaling in AD conditions, particularly in glial functions, are largely unknown. In this study, we investigated the potential benefits of MCR activation in AD. In APP/PS1 transgenic mice, we demonstrate that MCR activation mediated by the central administration of its agonist D-Tyr MTII substantially reduces Aβ accumulation, while alleviating global inflammation and astrocytic activation, particularly in the hippocampus. MCR activation prominently reduces the A1 subtype of reactive astrocytes, which is considered a key source of astrocytic neurotoxicity in AD. Concordantly, MCR activation suppresses microglial activation, while enhancing their association with amyloid plaques. The blunted activation of microglia may contribute to the reduction in the neurotoxic phenotypes of astrocytes. Importantly, transcriptome analysis reveals that MCR activation restores the impaired homeostatic processes and microglial reactivity in the hippocampus in APP/PS1 mice. Collectively, our findings demonstrate the potential of MCR signaling as therapeutic target for AD.

## Introduction

Alzheimer’s disease (AD) is among the most common neurodegenerative disorders and is characterized by the progressive deterioration of memory and cognitive functions. The major pathological hallmarks of AD are extracellular deposition of amyloid plaques due to the aggregation and accumulation of amyloid-beta (Aβ) peptides, intracellular deposition of hyperphosphorylated tau protein (termed “neurofibrillary tangles”), and progressive synaptic dysfunction and loss, which result in extensive neuronal loss^1–3^. The dysfunction of synapses in the hippocampus, the brain region associated with learning and memory, is an early event in AD progression^4–6^. Emerging evidence suggests that impairment of both neuronal and glial cells contributes to AD pathogenesis. As AD progresses, neuronal and glial dysfunctions lead to imbalanced Aβ production and clearance, consequently increasing Aβ burden. Such increased Aβ burden further alters the activation states of microglia and astrocytes^7,8^, which contribute to the loss of homeostasis in the central nervous system, further promoting AD pathology with chronic inflammation^9–13^.

Recent studies suggest the implications of G protein-coupled receptors (GPCRs) in the pathophysiology of AD. The members of the superfamily of GPCRs govern general physiological processes, including neural cell differentiation, synaptic transmission and plasticity, along with glial cell functions^14,15^. Accordingly, GPCRs are involved in several neurotransmitter systems associated with AD, namely the glutamatergic, serotonergic, adrenergic and peptidergic pathways, that are deregulated in AD^16^. Through these pathways, GPCRs modulate the production of Aβ peptides, and downstream plaque formation and Aβ clearance^17^. Specifically, dysregulated GPCR signaling is also implicated in the cognitive decline observed in AD^18,19^. Replenishing or activating GPCR signaling elicits multiple protective effects on synaptic functions, inflammatory pathways, adult neurogenesis, and the trafficking of APP (amyloid precursor protein)^20–22^. Therefore, understanding the deregulation and dysfunction of GPCRs involved in AD is essential for identifying therapeutic targets and intervention approaches for the disease.

The central melanocortin receptor (MCR) family of GPCRs consists of 5 members and has well-established roles in the regulation of energy homeostasis^23^, as well as anti-inflammatory and neuroprotective activities in diverse biological functions^24–26^. While MC3R and MC4R are both centrally expressed and abundant in the hypothalamus^27^, MC4R in particular is widely distributed throughout the central nervous system and prominently expressed in the hippocampus^28,29^. Alpha-melanocyte–stimulating hormone (α-MSH) is the endogenous agonist of the MCRs^27^, and is derived from the posttranslational cleavage of the precursor polypeptide, POMC (proopiomelanocortin)^30,31^. Recently, a functional POMC/MC4R microcircuit is identified in the hippocampus, wherein postsynaptic MC4R in the cornu ammonis 1 (CA1) region is activated by POMC neurons in the CA3 region^21^. Importantly, in the APP/PS1 transgenic mouse model of AD, impairment of the hippocampal α-MSH/MC4R circuit exacerbates precocious synaptic dysfunctions, whereas POMC overexpression or enhancement of MC4R signaling in the hippocampus rescues the synaptic plasticity deficits^21,32^, indicating its role in hippocampal synaptic transmission and plasticity. Furthermore, studies showed that peripheral administration of a synthetic melanocortin analogue in AD transgenic mouse models rescues synaptic dysfunction and ameliorates AD-related neurodegenerative signatures, including Aβ deposition, neuronal loss, and inflammation^21,33,34^. Furthermore, peripheral administration of a synthetic melanocortin analogue in AD transgenic mouse models resulted in reduction in Aβ deposition and neuronal loss in the hippocampus and isocortex^33,34^. However, the effect of melanocortin signaling on the multiple neurodegenerative signatures of AD, particularly the glial activation and inflammatory shift underlying AD pathogenesis, remains to be examined.

In this study, we examined whether and how MCR activation exerts beneficial effects on AD pathophysiology. We showed that overexpressing the MCR agonist in the hippocampus or activating the receptor by its agonist D-Tyr MTII (D-Tyr) reduces Aβ level and its deposition in APP/PS1 transgenic mice. MCR activation reduces global neuroinflammation and astrocytic activation. Intriguingly, MCR activation in APP/PS1 mice led to the drastic reduction of the neurotoxic A1 subtype of reactive astrocytes, which is the major astrocytic subtype that mediates astrocytic toxicity in AD. Concordant with the understanding that A1 neurotoxic astrocytes are induced by classically activated neuroinflammatory microglia, MCR activation is demonstrated to reduce the activation of microglia but enhances their recruitment to amyloid plaques (which may facilitate amyloid clearance). Transcriptome analysis revealed that direct stimulation of hippocampal slices from APP/PS1 mice with D-Tyr was able to rescue the altered molecular pathways, including microglial activation pathway and cellular homeostasis. Together, these findings support the beneficial actions of MCR activation and their molecular basis in AD pathogenesis.

## Materials and methods

### Animals

APP/PS1 (i.e., B6C3-Tg[APPswe, PSEN1dE9]85Dbo) double-transgenic mice were generated by incorporating a human/murine APP construct bearing the Swedish double mutation and the exon 9-deleted *PSEN1* mutation (*APPswe* + *PSEN1/dE9*)^35^. The transgenic mouse line was obtained from the Jackson Laboratory. Genotypes were verified by PCR analysis of tail or ear biopsies.

All mice were housed at the Animal Care Facility of the Hong Kong University of Science and Technology (HKUST). All animal experiments were conducted in accordance with guidelines and regulations that were approved by the Animal Ethics Committee of HKUST, and in compliance with the ARRIVE guidelines. Four to five mice of the same sex were housed per cage under a 12/12-h light–dark cycle and water and standard chow diet ad libitum. Male mice were used for all experiments. Mice were assigned to experimental groups according to body weight. Sample sizes were decided on the basis of experience with similar types of experiments.

### In vivo experiments

Osmotic pump infusion was performed on 6–7-month-old APP/PS1 mice as previously described^9–13^. Alzet mini-osmotic pumps (model 1004) were set at an infusion rate of 0.11 μL/h and implanted for 28 days. The pumps were filled with [d-Tyr^4^]-melanotan II (d-Tyr; 043–29, Phoenix Pharmaceuticals) in Dulbecco’s phosphate-buffered saline (DPBS) or DPBS as control (i.e., vehicle; Veh), and connected to the right lateral ventricle of the mice. The drugs treatments were administered at 2.4 nmol/day. The mice were euthanized with isoflurane after 28 days.

Prior to commencement of the stereotaxic surgery, mice were anesthetized with 2% isoflurane while immobilized on a stereotaxic frame. The skull was exposed through a small incision, and craniotomy was performed for injection using a 2-μL Hamilton syringe (World Precision Instruments) with a 33-gauge beveled metal needle. The unit is connected to a microsyringe infusion pump and controller (53,311; Stoelting Company). Virus was infused at 100–150 nL/min. At the completion of infusion, the needle was retained at the injection site for 10 min and withdrawn slowly. All stereotaxic coordinates are measured relative to the bregma. Prior to recovery on a heat pad, the animals were subjected to subcutaneous injection with antibiotics (penicillin 10,000 IU and streptomycin 10,000 μg/mL at 10 mL/kg), and the incision was closed with sutures with topical application of penicillin.

To restore POMC expression, 7–8-month-old APP/PS1 mice were co-injected with the adeno-associated viruses (AAVs) *AAV*_*2/9*_*-EF1α-DIO-POMC* (*AAV-DIO-POMC*) (7 × 10^12^ TU/mL; a gift from Eriika Savontaus, the University of Turku) and *AAV*_*2/9*_*-POMC-Cre* (*AAV-POMC-Cre*) (7 × 10^12^ TU/mL; Shenzhen Institutes of Advanced Technology, Chinese Academy of Sciences). The viruses were diluted 300 fold at 1:1 v/v. One μL of the virus mixture was then injected into the dorsal hippocampal CA2/CA3 region. Control group was co-injected with *AAV-POMC-Cre* and *AAV*_*2/5*_*-hSyn-DIO-mCherry* (*AAV-DIO-mCherry*) (6.4 × 10^12^ TU/mL; Vector Core at the University of North Carolina). The mice were euthanized with isoflurane after 28 days.

### Immunohistochemical analysis

Immunohistochemical staining was performed as previously described^9–13^. For fixation of the brain, mice were first anesthetized with isoflurane, and brains were fixed by transcardial perfusion with 0.9% saline followed by ice-cold 4% paraformaldehyde in 0.1 M PBS (pH 7.4). The brains were collected promptly and post-fixed in 4% paraformaldehyde for 6 h prior to overnight cryoprotection in 30% D-glucose in PBS. Free-floating coronal brain cryosections were prepared at 30-μm thickness, and preserved in cryoprotectant (30% glycerol and 30% ethylene glycol in PBS) at − 20 °C storage until use. Brain sections were generously washed in 0.2% PBS–Triton X-100 (PBST) prior to immunostaining procedures.

For staining of amyloid plaque deposition, antigen retrieval was performed by incubation of the sections, first in 70% formic acid in H_2_O for 5 min, then in 3% H_2_O_2_ in H_2_O for 15 min to inhibit the activity of endogenous peroxidase. The sections were blocked with 5% horse serum in PBST for 2 h, followed by immunostaining with 6E10 antibody (anti-Aβ_1–16_; 803,015, BioLegend) at 1:1,000 dilution in PBST at 4 °C overnight. Sections were then incubated with biotinylated horse anti-mouse IgG antibody (Life Technologies) at 1:1,000 dilution for 2 h at room temperature. A DAB Peroxidase Substrate Kit (SK-4100, Vector Laboratories) with 3,3′-diaminobenzidine as the chromogen was used for development. For immunofluorescence staining of amyloid plaque deposition, sections were subjected to antigen retrieval and then a 2-h blocking step, followed by incubation with 6E10 antibody overnight, and subsequently with Alexa Fluor-conjugated IgG (Life Technologies) for 2 h.

Imaging was performed by bright-field microscopy on a Leica DM6000 B compound microscope system. Analysis of the amyloid plaque areas in the hippocampus and cortex in the brain sections were conducted using the *Analyze Particles* function in ImageJ (version 1.61). The entire hippocampal and neocortical regions were used for analysis. The analysis included 4 brain sections per mouse at 30-µm intervals, and the average percentage of the hippocampal or cortical area occupied by amyloid plaques was measured.

To examine the colocalization of amyloid plaques with astrocytes and C3 (complement component 3), the brain sections were treated with Mouse on Mouse Blocking Reagent (MKB-2213, Vector Laboratories) prior to blocking step in order to inhibit endogenous mouse immunoglobulins. This additional step was omitted from the immunostaining procedures for the colocalization of amyloid plaques with microglia. Free-floating brain sections for both assessments were subsequently blocked with 1% bovine serum albumin, 4% goat serum, and 0.3% PBST for 2 h at room temperature. To assess microglial expression, sections were incubated overnight with the ionized calcium-binding adapter molecule 1 (Iba1) antibody (019–19,741, Wako) at 1:1,000 dilution and the 4G8 antibody (anti-Aβ_17–24_; 800,718, BioLegend) at 1:500 dilution at 4 °C. To assess glial fibrillary acidic protein (GFAP) and C3 immunoreactivity in astrocytes, sections were incubated overnight with GFAP antibody (AB5804, Millipore) at 1:2,000 dilution, C3 antibody (Aβ11862, Abcam) at 1:50 dilution, and 4G8 antibody at 1:500 dilution at 4 °C. Fluorescence immunostaining imaging was performed using the Leica TCS SP8 confocal system. ImageJ was employed for the quantification of the density of Iba1^+^ cells and association with amyloid plaques, the intensity and areas of GFAP expression, and the colocalization GFAP with C3^+^ cells. Three brain sections 100–150 μm apart along the anteroposterior from each mouse for each assessed area (i.e., the cortex, CA1 region, or CA3 region) were analyzed. To assess the degree of colocalization of amyloid plaques with microglia, three to five Iba1^+^ microglia-associated amyloid plaques from each mouse were selected for analysis. The selected amyloid plaques were of comparable size between Veh- and d-Tyr–treated APP/PS1 groups.

### Amyloid-beta extraction, western blotting and ELISA

Aβ was extracted from the soluble and insoluble fractions of the mouse hippocampus and cortex tissues^35,36^. In brief, frozen brain tissues were lysed in lysis buffer with various protease inhibitors^10^. Accordingly, the mouse hippocampi and cortices were subsequently homogenized in buffer comprising 250 mM sucrose, 20 mM Tris-hydrochloride (pH 7.4), 1 mM ethylene diamine tetra acetic acid, 1 mM ethylene glycol tetraacetic acid, and protease inhibitor cocktail (Sigma-Aldrich). Soluble Aβ was sequentially extracted by diethylamine (soluble) followed by formic acid (insoluble). The protein levels of soluble and insoluble Aβ were determined by western blot analysis with 6E10 antibody. The levels of soluble Aβ_x–40_ (KHB3481) and Aβ_x–42_ (KHB3441) were assessed by ELISA using commercial ELISA kits (Thermo Scientific).

To assess inflammatory factors, the protein levels of Iba1 and GFAP (anti-GFAP; 3670S, Cell Signaling Technology) were determined by western blot analysis. Densitometric quantification of protein band intensity from western blot analysis was performed using ImageJ. The Quantikine ELISA kit (MLB00C, R&D Systems) was used to determine the protein level of interleukin-1 beta (IL-1β).

### Quantitative RT-PCR analysis

For quantitative RT-PCR procedures, TRIzol (Invitrogen) was used to extract RNA from the hippocampal and cortical regions. The RNA was subsequently purified using the NucleoSpin RNA Clean-up kit (Macherey–Nagel). The BioDrop μLITE microvolume spectrophotometer was employed to determine RNA purity and concentration. The PrimeScript RT-PCR Kit (TaKaRa) was used for the reverse transcription of equivalent amounts of RNA for cDNA synthesis. PCR amplification and quantitative real-time detection of PCR products were conducted using the TaqMan gene expression assay (Applied Biosystems) and the Premix Ex Taq qPCR assay (TaKaRa), respectively. Two μL cDNA product was input in a total reaction volume of 20 μL. The values representing mRNA expression were normalized to that of beta (β)-actin. The TaqMan probes used are as follows: *Aif1* (Mm00479862_g1), *Gfap* (Mm01253033_m1), *Il1β* (Mm01336189_m1), *Il6* (Mm00446190_m1), *Icam1* (Mm00516023_m1), *Itgam* (Mm00434455_m1), *Chmp3* (Mm00850329_s1), *Zfp579* (Mm02766069_s1) and *β-actin* (Mm02619580_g1).

### Microarray analysis of acute hippocampal slices

Acute hippocampal slices, 300-μm-thick, were prepared from wild-type (WT) and APP/PS1 mice at 6 months of age. The sections were recovered in artificial cerebrospinal fluid for 2 h, and subjected to treatments with either PBS (Veh) or 1 μM D-Tyr for 2 h. The entire hippocampal region from the slices were then collected and RNA extracted for transcriptome analysis by RNA-sequencing (Novaseq). Transcript-level expression analysis was performed on Mouse brain RNA-seq data. The bioinformatics QC was generated using FastQC and RSeQC. The transcript-level expression analysis had followed the protocol described by Pertea et al., 2016^37^. The protocol used the following software for analysis: HISAT2, StringTie, then DESeq2. The pipeline to run the analysis was generated by Snakemake workflow management system. Differentially expressed genes (DEGs) were functionally annotated by Gene Ontology (GO) and protein–protein interaction analysis of the DEGs were performed by Search Tool for the Retrieval of Interacting Gene (STRING).

### Statistical analysis

Statistical analysis adopted in the present work was modified from previously description^9–13^. The researchers who conducted the immunohistochemical and transcript expression analyses were blinded to both the mouse genotypes and treatment conditions. All data are presented as arithmetic mean ± SEM. Differences amongst mouse groups of various genotypes or treatment conditions were evaluated by unpaired Student’s *t-*tests or one-way ANOVA combined with Bonferroni post hoc analysis where appropriate. All statistical analyses were performed using GraphPad Prism 8.4.2 (GraphPad Software). The level of statistical significance was defined at *p* < 0.05.

## Results

### Restoration of POMC expression in hippocampal CA3 region in APP/PS1 mice reduces amyloid-beta pathology

**Figure 1:**
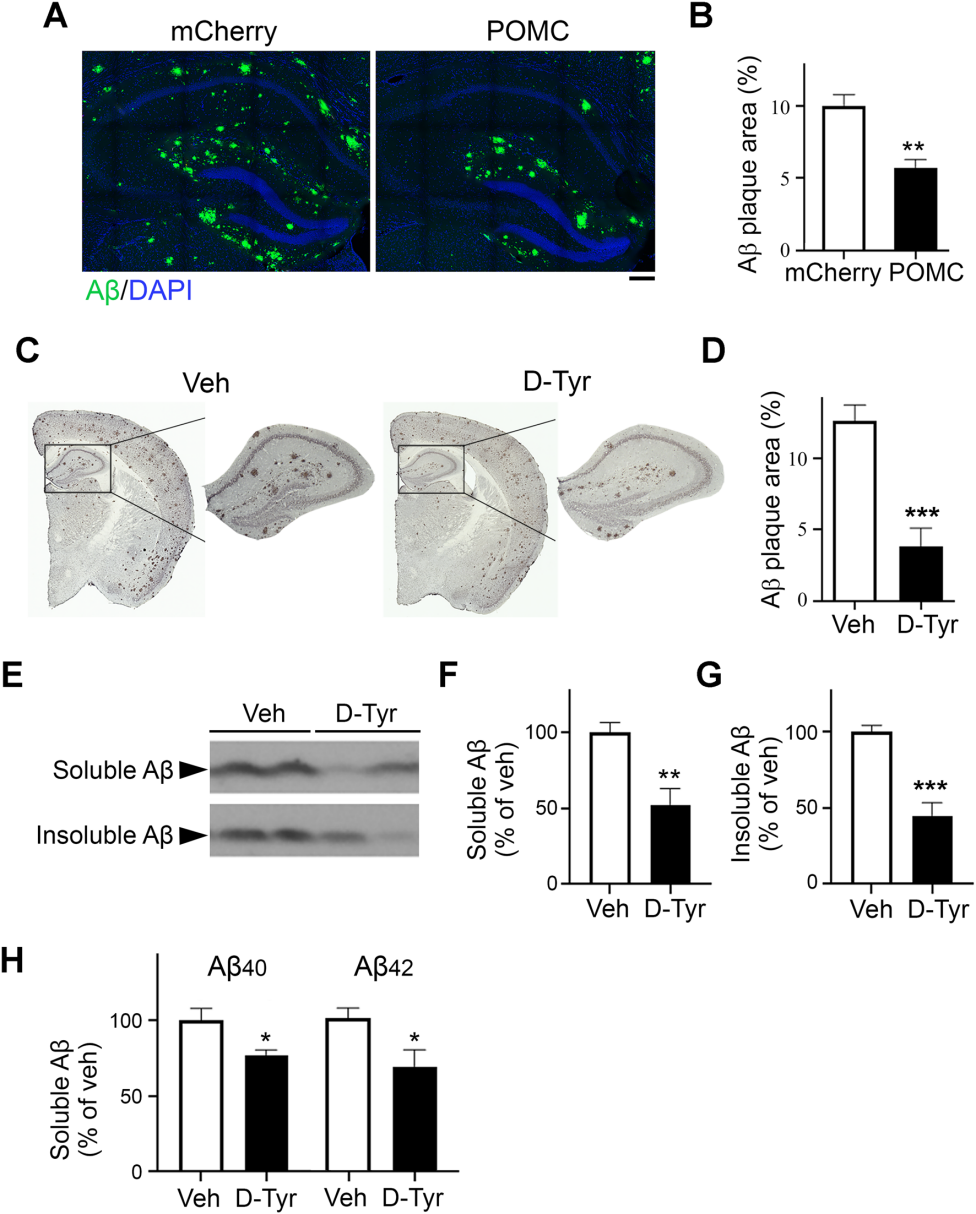
Activation of POMC/MCR signaling ameliorates amyloid pathology in the hippocampus in APP/PS1 mice. (**A**,**B**) The restoration of POMC expression in the CA3 region reduces the amyloid plaque area in the hippocampus in APP/PS1 mice. Representative fluorescence micrographs (**A**) and quantification (**B**) of 6E10-stained amyloid plaques in the hippocampus on coronal brain sections of APP/PS1 mice following intrahippocampal co-injection of Cre-dependent AAV-POMC-Cre/AAV-DIO-POMC (POMC) versus AAV-POMC-Cre and AAV-DIO-mCherry (mCherry) as a control (*n* = 5 mice per group, 3 sections per mouse at 100–150-µm intervals). Amyloid plaques are shown in green, and nuclei are shown in blue. Scale bar = 100 µm. (**C**,**D**) Chronic d-Tyr administration reduced amyloid plaque deposition in APP/PS1 mice. Representative images (**C**) and quantification (**D**) of 6E10-stained amyloid plaques in the hippocampus on coronal brain sections of APP/PS1 mice following treatment with chronic d-Tyr versus control (Veh) (*n* = 8–9 mice per group, 4 sections per mouse at 30-µm intervals). Rectangles denote the hippocampal area in the insets. Scale bar = 1 mm. (**E**–**G**) Chronic d-Tyr administration reduced soluble and insoluble Aβ contents. Representative western blot (**E**) and quantification of soluble (**F**) and insoluble (**G**) Aβ levels in hippocampal homogenates from APP/PS1 mice (*n* = 8–9 mice per group). Full-length blots are presented at the end of Supplemental Information. As the blots were cut prior to hybridization with antibodies, membrane edges were outlined with solid black lines. (**H**) Quantitative assessment (i.e., ELISA) of the relative levels of Aβ_x–40_ and Aβ_x–42_ isomers in the soluble fraction from the hippocampus of APP/PS1 mice (*n* = 4–5 mice per group). Data are the mean ± SEM of all mice from each group examined (*p < 0.05, **p < 0.01, ***p < 0.001 for chronic d-Tyr vs. Veh or POMC vs. mCherry injection; Student’s t-test).

Given that the functional POMC/MC4R microcircuit in the hippocampal CA3–CA1 circuit is perturbed and that POMC neurons are vulnerable to Aβ in the APP/PS1 transgenic mouse model of AD, restoration of this circuit can rescue the synaptic plasticity impairment in such transgenic mice^21^. Therefore, in the present study, we further examined whether restoring the hippocampal expression of POMC positively affects Aβ deposition pathology. Accordingly, we overexpressed POMC protein in POMC neurons in the CA3 region in APP/PS1 mice at 8 months of age, when amyloid plaque deposition is extensive in the brain. Overexpression of POMC in the CA3 region approximately halved the amyloid plaque area in the hippocampus (Fig. 1A,B). No such reduction was observed in the cortex in the same mice (data not shown), indicating that POMC-expressing CA3 neurons exert a relatively localized effect on the amelioration of amyloid plaque deposition. These results collectively suggest that the enhancement of melanocortin signaling in the hippocampus alleviates amyloid pathology.

### Melanocortin receptor activation ameliorates amyloid pathology in APP/PS1 mice

Restoration of the CA3–POMC/CA1–MC4R microcircuit alleviated hippocampal synaptic plasticity impairment and reduced Aβ deposition in APP/PS1 mice (Fig. 1)^21^, suggesting that replenishing melanocortin signaling in the brain likely exerts beneficial effect. Corroborating previous findings that MCR activation reduces amyloid deposition^33,34^, we showed that delivery of the specific MCR agonist, d-Tyr, into 6-month-old APP/PS1 mice (when Aβ deposits begin to develop^35^) significantly reduced (~ 70%) 6E10-immunostained amyloid plaques in the hippocampus compared to vehicle-treated controls (Fig. 1C,D). Besides decreasing the level of insoluble Aβ (Fig. 1E,G), d-Tyr administration significantly decreased the hippocampal level of soluble Aβ (Fig. 1E,F), which is believed to contain the major synaptotoxic Aβ species. Concordantly, after d-Tyr administration, the level of Aβ_x–40_, which is suggested to account for approximately half of all Aβ isomers^4–6^, was markedly reduced in the soluble fraction of the hippocampus from APP/PS1 mice (Fig. 1H). The level of Aβ_x–42_, the isomer most prone to precipitation and thus the most toxic to synaptic plasticity^4–6^, also decreased significantly (Fig. 1H).

Similarly, APP/PS1 mice treated with d-Tyr exhibited significantly decreased amyloid plaque deposition in the neocortex (Supplemental Fig. 1A,B) as well as similar decreases in soluble and insoluble Aβ levels in the cortex (Supplemental Fig. 1C–E). Interestingly, the level of Aβ_x–42_ but not Aβ_x–40_ was significantly reduced in the cortex (Supplemental Fig. 1F). These results indicate that chronic MCR activation improves amyloid pathology in the hippocampus and cortex in APP/PS1 mice potentially with different impacts in specific brain regions.

### Chronic melanocortin receptor activation ameliorates neuroinflammation in APP/PS1 mice

**Figure 2:**
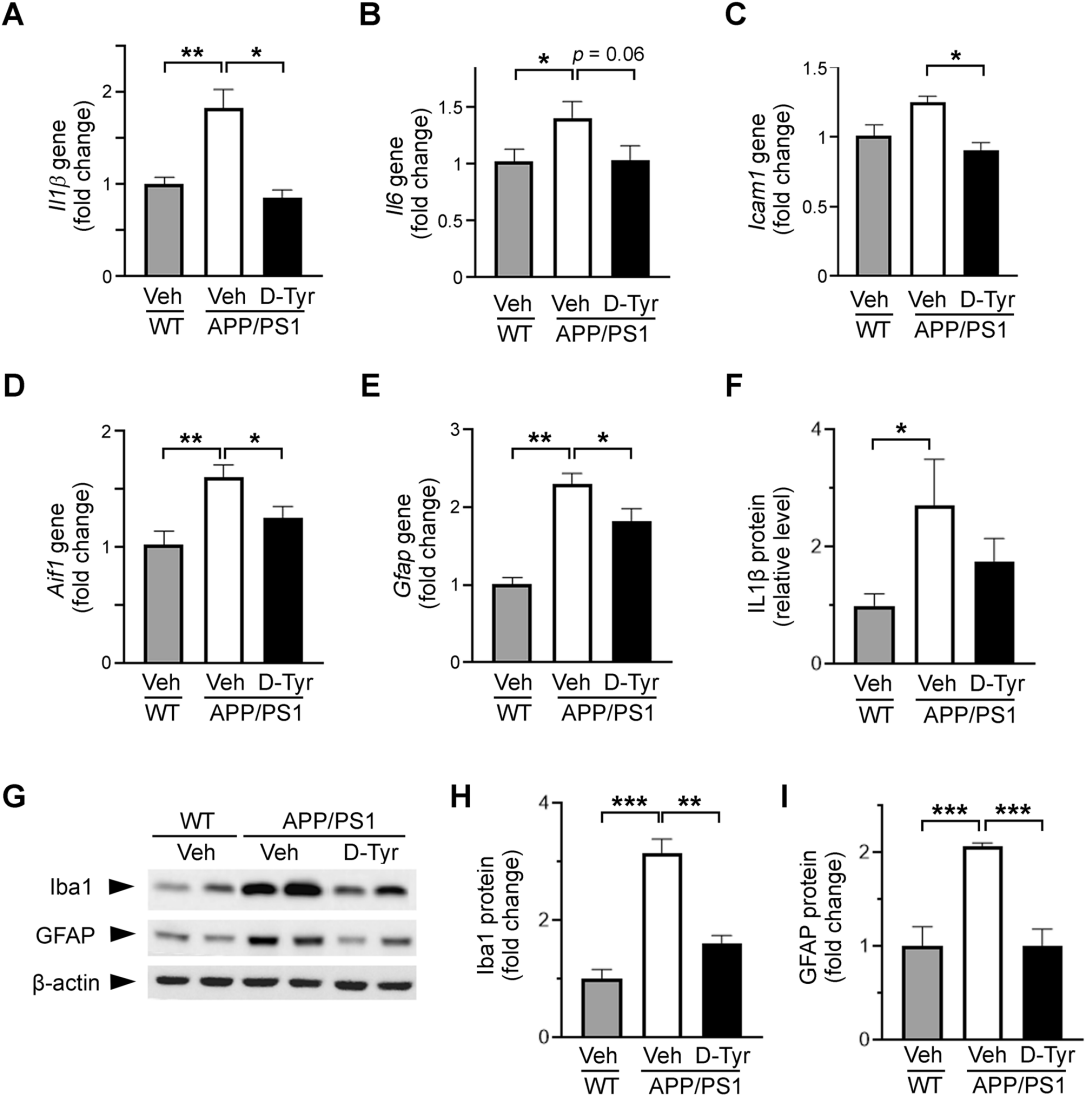
Activation of melanocortin signaling alleviates neuroinflammation in the hippocampus in APP/PS1 mice. (**A**–**E**) Quantitative PCR showing the regulation of inflammatory genes in the hippocampus in WT mice treated with control (Veh) and APP/PS1 mice treated with d-Tyr versus control (Veh). All measurements are normalized to the level of β-actin and presented as the fold expression relative to the average of the WT-Veh group. Transcript levels of the pro-inflammatory cytokines *Il1β* (**A**) and *Il6* (**B**), the anti-inflammatory marker *Icam1* (**C**), the microglial marker *Aif1* (**D**), and the astrocyte marker *Gfap* (**E**) (*n* = 5 mice per group). Quantitative assessment (i.e., ELISA) of the relative level of IL-1β (**F**) in the hippocampus (*n* = 5 mice per group). Representative western blot (**G**) and quantification of Iba1 (**H**) and GFAP (**I**) protein levels in hippocampal homogenates from WT and APP/PS1 mice (*n* = 4 mice per group). Full-length blots are presented at the end of Supplemental Information. As the blots were cut prior to hybridization with antibodies, membrane edges were outlined with solid black lines. Data are the mean ± SEM of all mice from each group (*p < 0.05, **p < 0.01, ***p < 0.001; one-way ANOVA with the Bonferroni post hoc test).

The accumulation of amyloid plaque deposition is closely associated with the extent of neuroinflammation in AD progression^3,9,10^. Consistent with the chronic neuroinflammation observed in AD transgenic mouse models including APP/PS1 mice^3,38^, 7-month-old APP/PS1 mice exhibited substantial increase in the transcript levels of *Il1β* and *Il6* (major pro-inflammatory factors) and intercellular adhesion molecule 1 (*Icam1*) (an anti-inflammatory cytokine) in the hippocampus (Fig. 2A–C) and cortex (Supplemental Fig. 2A–C) compared to their WT counterparts. Notably, chronic d-Tyr administration abolished the elevated transcript levels of these pro- and anti-inflammatory factors in the hippocampus in APP/PS1 mice (Fig. 2A–C). Interestingly, d-Tyr administration suppressed the elevated transcript levels of *Il1β* and *Icam1* in the cortex but did not affect the upregulated expression of *Il6* (Supplemental Fig. 2A–C). Concordant with the transcriptional alteration, the protein expression of IL-1β measured by ELISA resembled the relative changes between the experimental groups in both the hippocampus (Fig. 2F) and cortex (Supplemental Fig. 2F).

Given that glial cells play a pivotal role in the elicitation of chronic neuroinflammation and aggravation of AD pathology^9,38,39^, we examined whether and how replenishment of the melanocortin system affects the glial phenotypes in AD. In APP/PS1 mice, the transcript levels of allograft inflammatory factor 1 (*Aif1*) (a microglia/macrophage marker gene encodes Iba1) and *Gfap* (an astrocyte marker), which serve as indicators of the reactivity of glial cells, were significantly upregulated in both the hippocampus (Fig. 2D,E) and neocortex (Supplemental Fig. 2D,E). Furthermore, d-Tyr administration significantly decreased the transcript levels of both *Gfap* and *Aif1* in the hippocampus in APP/PS1 mice (Fig. 2D,E) but only that of *Gfap* in the cortex (Supplemental Fig. 2D,E). Western blot analysis showed comparable changes in the relative protein expressions of both Iba1 and GFAP between treatment groups in the hippocampus (Fig. 2G–I) and cortex (Supplemental Fig. 2G–I). Taken together, these findings suggest that d-Tyr differentially alleviates the inflammatory tone in different brain regions in APP/PS1 mice; it specifically alleviates microglial activation in the hippocampus but reduces global astrocyte activation.

### Melanocortin receptor activation alleviates astrocyte reactivity in the hippocampal CA1 region in APP/PS1 mice

**Figure 3:**
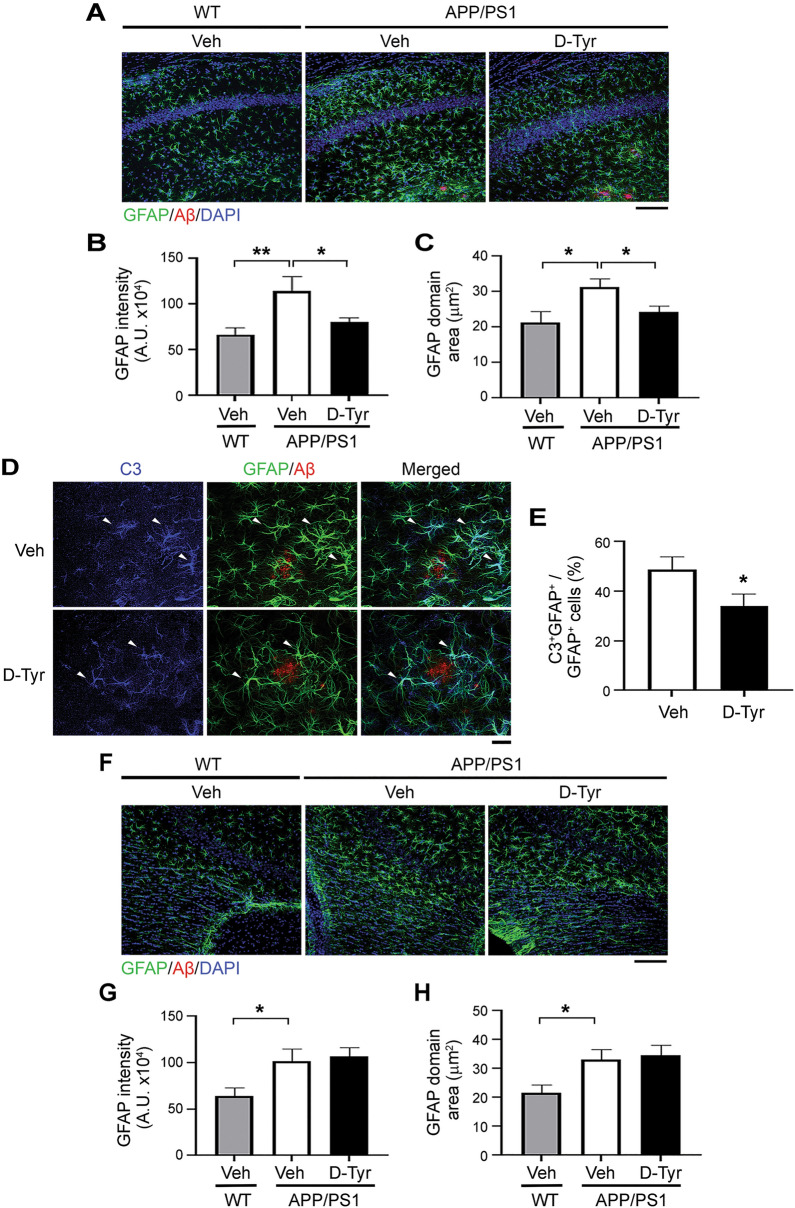
Activation of melanocortin signaling ameliorates astrocyte reactivity in the hippocampal CA1 region in APP/PS1 mice. (**A**–**C**) Chronic d-Tyr administration reduced GFAP expression in astrocytes in the hippocampal CA1 region in APP/PS1 mice. Representative immunostaining (**A**), immunoreactivity in arbitrary units (A.U.) (**B**), and average domain areas (**C**) of GFAP in each astrocyte in the hippocampal CA1 region on coronal brain sections of WT mice treated with control (Veh) and APP/PS1 mice treated with chronic d-Tyr versus Veh. Scale bar = 100 µm (*n* = 9 mice per group; *p < 0.05, **p < 0.01, one-way ANOVA with the Bonferroni post hoc test). (**D**,**E**) Chronic d-Tyr administration reduced the C3^+^ (complement component 3) A1 subtype of reactive astrocytes. Representative images (**D**) and quantification (**E**) of C3^+^GFAP^+^ co-labeled astrocytes (white arrows) in the hippocampal CA1 region in APP/PS1 mice treated with chronic d-Tyr versus Veh. Scale bar = 20 µm (*n* = 9 mice per group; **p < 0.01; Student’s *t*-test). Chronic d-Tyr–mediated activation of MCR signaling did not affect the reactivity of astrocytes in the hippocampal CA3 region in APP/PS1 mice. (**F**–**H**) Chronic d-Tyr administration did not alter GFAP expression in astrocytes in the hippocampal CA3 region. Representative immunostaining (**F**), immunoreactivity in A.U. (**G**), and average domain areas (**H**) of GFAP of each astrocyte in the hippocampal CA3 region on coronal brain sections of WT mice treated with Veh and APP/PS1 mice treated with chronic d-Tyr versus Veh. Scale bar = 100 µm (*n* = 9 mice per group; *p < 0.05 for WT vs. APP/PS1 mice receiving Veh treatment; one-way ANOVA with the Bonferroni post hoc test).

We subsequently examined the specific effects of chronic activation of melanocortin signaling on astrocyte activation in AD transgenic mouse models. Astrocytes, the quintessential immune effector cells that govern Aβ clearance^38,40,41^, are activated in APP/PS1 mice, as reflected by increased GFAP expression^3,42^. Consistent with previous findings, we observed elevated GFAP immunoreactivity in larger domain areas in the hippocampal CA1 (Fig. 3A–C) and CA3 (Fig. 3F–H) regions as well as the cortex (Supplemental Fig. 3A–C) in 7-month-old APP/PS1 mice. Administration of d-Tyr prominently reverted the elevated astrocyte reactivity in APP/PS1 mice, as indicated by a significant decrease in GFAP intensity in diminished domain areas in the hippocampal CA1 region (Fig. 3A–C) and cortex (Supplemental Fig. 3A–C). However, this effect was not observed in the hippocampal CA3 region (Fig. 3F–H).

Recent studies suggest that a subtype of reactive astrocytes termed neurotoxic A1 astrocytes, in which C3 is highly upregulated, constitute the main astrocytes that contribute to the death of neurons in neurodegenerative disorders such as AD^11,43^. GFAP and C3 co-staining revealed that nearly half of GFAP^+^ astrocytes in the hippocampus (Fig. 3D) and cortex (Supplemental Fig. 3D) in APP/PS1 mice were C3-positive (i.e., C3^+^GFAP^+^ A1 reactive astrocytes). In addition, d-Tyr administration reduced the proportion of C3^+^GFAP^+^ astrocytes in the hippocampal CA1 region by 30% (Fig. 3D,E) and the cortex by 23% (Supplemental Fig. 3D,E). However, no such decrease was observed in the CA3 region (data not shown). These results collectively suggest that d-Tyr administration either inhibits the formation of C3^+^GFAP^+^ astrocytes or converts the neurotoxic state of this subtype of astrocytes.

### Enhancement of melanocortin signaling alleviates microglial reactivity in the hippocampus in APP/PS1 mice

**Figure 4:**
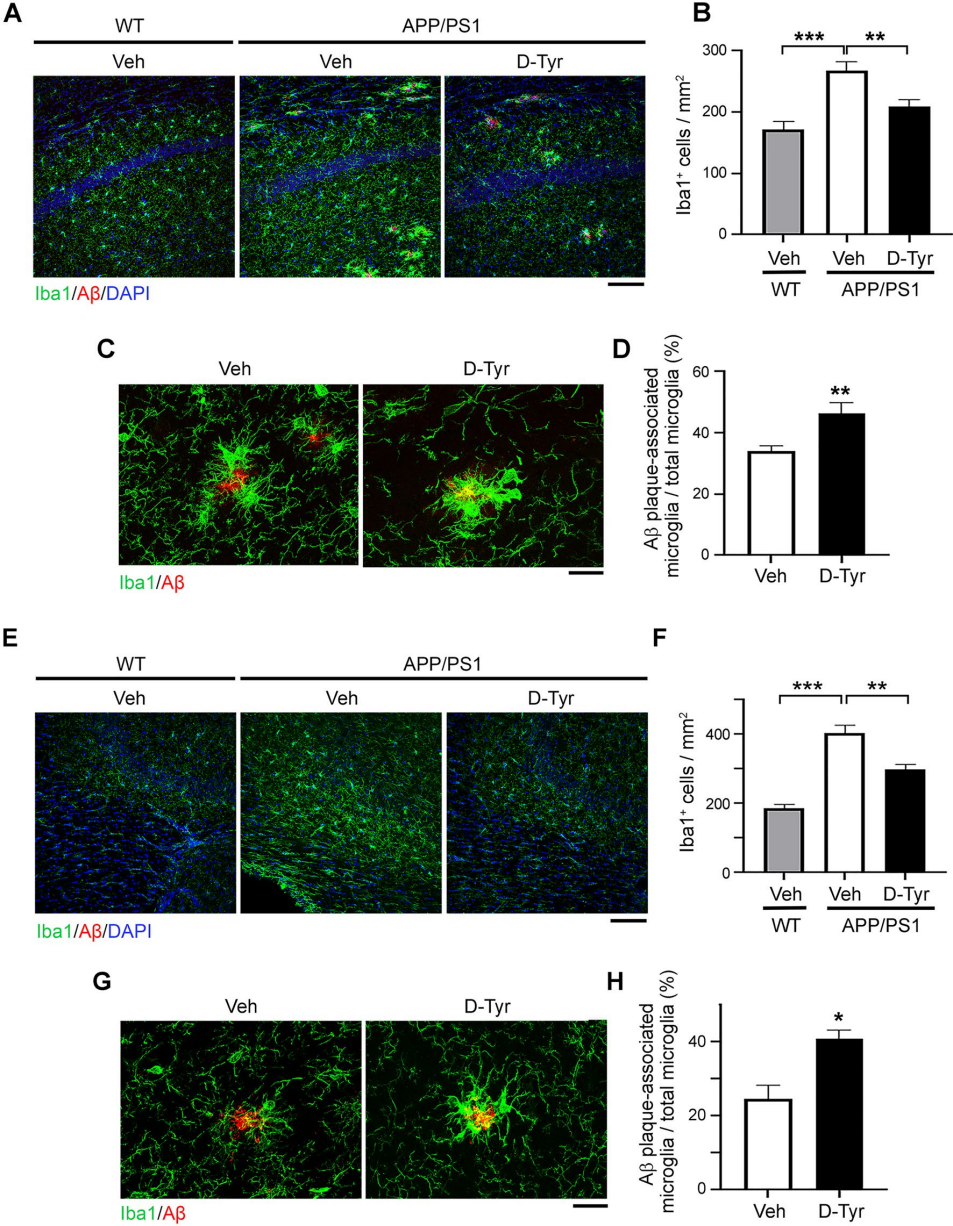
Activation of melanocortin signaling ameliorates microglial reactivity in the hippocampus in APP/PS1 mice. (**A**–**D**) Chronic d-Tyr administration ameliorated microglial reactivity in the hippocampal CA1 region in APP/PS1 mice. Representative immunostaining (**A**) and quantification of the density (**B**) of microglia (labeled with Iba1) in the hippocampal CA1 region on coronal brain sections of WT mice treated with control (Veh) and APP/PS1 mice treated with chronic d-Tyr versus Veh. Scale bar = 100 µm (*n* = 9 mice per group; **p < 0.01, ***p < 0.001; one-way ANOVA with the Bonferroni post hoc test). (**C**–**D**) Chronic d-Tyr administration increased the association of microglia with amyloid plaques in the hippocampal CA1 region in APP/PS1 mice. Representative images (**C**) and quantification (**D**) of amyloid plaque-associated Iba1^+^ microglia in the hippocampal CA1 region in APP/PS1 mice subjected to treatment with chronic d-Tyr versus Veh. Scale bar = 20 µm (*n* = 8 mice per group; **p < 0.01; Student’s t-test). (**E**–**H**) Activation of MCR signaling alleviated microglial reactivity in the hippocampal CA3 region in APP/PS1 mice. Representative immunostaining (**E**) and quantification of the density (**F**) of microglia (labeled with Iba1) in the hippocampal CA3 region on coronal brain sections of WT mice treated with Veh and APP/PS1 mice treated with chronic D-Tyr versus Veh. Scale bar = 100 µm (*n* = 9 mice per group; **p < 0.01, ***p < 0.001; one-way ANOVA with the Bonferroni post hoc test). (**G**, **H**) Chronic d-Tyr administration increased the association of microglia with amyloid plaques in the hippocampal CA3 region in APP/PS1 mice. Representative images (**G**) and quantification (**H**) of amyloid plaque-associated Iba1^+^ microglia in the hippocampal CA3 region in APP/PS1 mice subjected to treatment with chronic d-Tyr versus Veh. Scale bar = 20 µm (*n* = 8 mice per group; *p < 0.05; Student’s t-test).

Considering the observed reduction of C3^+^GFAP^+^ A1 astrocytes due to d-Tyr administration (Fig. 3) and that the A1 subtype of reactive astrocytes is induced by activated microglia^11^, we investigated the effect of d-Tyr administration on the activity of microglia. Concordant with heightened central inflammatory signature of AD pathology^9,39^, microglia density was substantially higher in the hippocampal CA1 (Fig. 4A,B) and CA3 (Fig. 4E,F) regions as well as the cortex (Supplemental Fig. 4A,B) in APP/PS1 mice when compared with their WT counterparts at 7 months of age. Notably, in APP/PS1 mice, d-Tyr administration significantly reduced microglial density in the hippocampal CA1 (Fig. 4A,B) and CA3 (Fig. 4E, F) regions but not the cortex (Supplemental Fig. 4A–B).

In Aβ clearance by microglia, microglia are recruited to amyloid plaques before becoming activated and phagocytosing Aβ^44,45^. Therefore, in APP/PS1 mice, amyloid plaques are typically surrounded by clusters of microglia in the hippocampus (Fig. 4C,G) and cortex (Supplemental Fig. 4C). Notably, d-Tyr administration significantly increased the proportion of microglia associated with amyloid plaques in the hippocampal CA1 (Fig. 4C,D) and CA3 (Fig. 4G,H) regions in APP/PS1 mice, suggesting that MCR activation enhances the proximity of microglia and hence their recruitment to Aβ. Meanwhile, no such alterations were observed in the cortex in APP/PS1 mice (Supplemental Fig. 4C,D). Thus, these results demonstrate that melanocortin/MCR signaling activation specifically regulates the microglial phenotypes and dynamics in the hippocampus in AD mouse models.

### Molecular action of hippocampal melanocortin signaling in the amelioration of Alzheimer’s disease pathology

**Figure 5:**
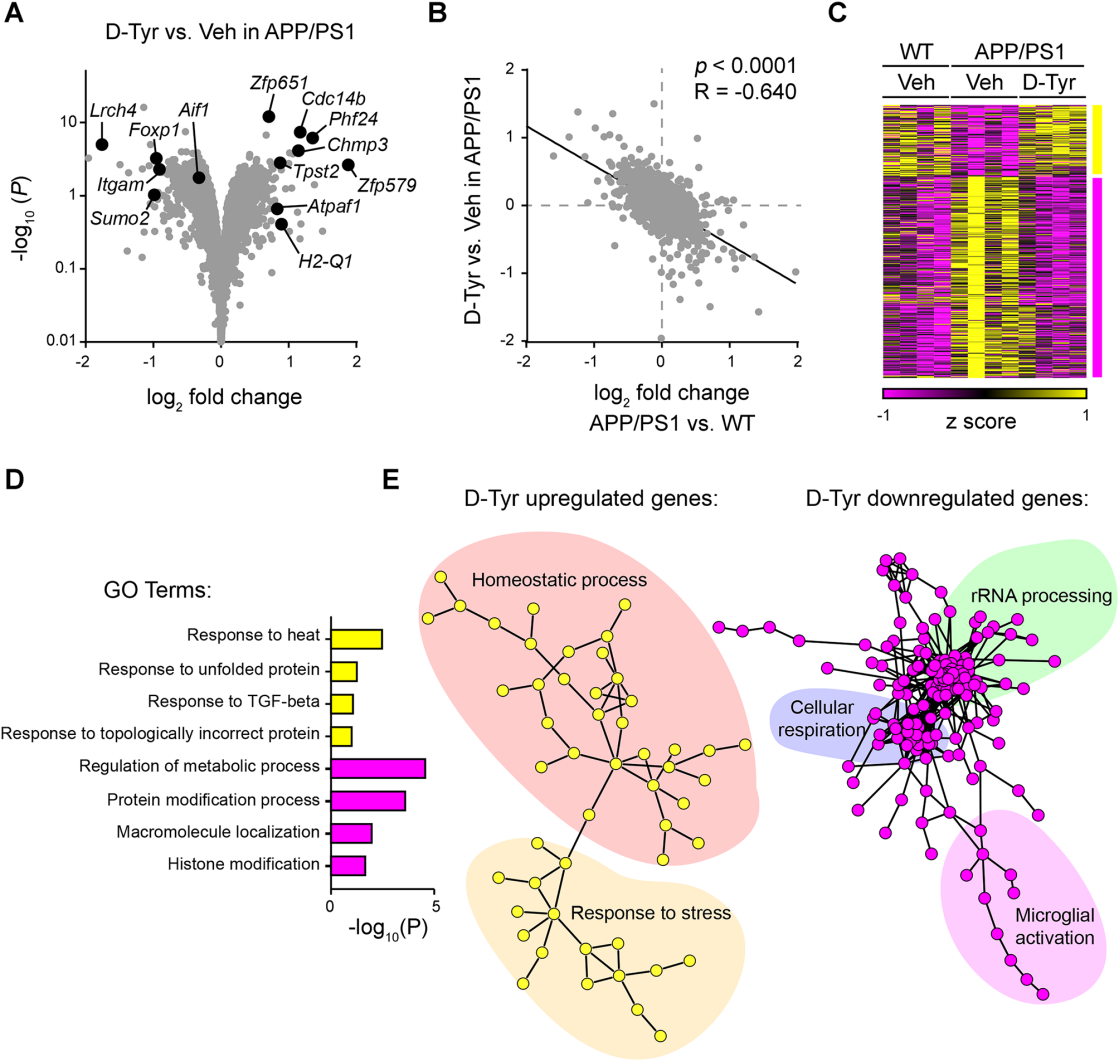
Administration of d-Tyr restores the dysregulated transcriptomic signature in APP/PS1 mice. (**A**) Volcano plot showing the differentially expressed genes (DEGs) upon d-Tyr administration in APP/PS1 mice. (**B**–**C**) d-Tyr administration reverted the transcriptomic changes in APP/PS1 mice. (**B**) Correlations between global transcriptomic changes of d-Tyr- versus vehicle (Veh)-treated APP/PS1 mice and APP/PS1 versus WT mice (r =  − 0.64, *p* < 0.0001; linear regression). (**C**) Heatmap showing the expression levels of DEGs in d-Tyr- versus Veh-treated APP/PS1 mice, and Veh-treated WT mice. (**D**–**E**) Pathway analysis demonstrating the functional associations of d-Tyr–induced DEGs with stress response, rRNA processing, cellular respiration, and microglial activation. (**D**) Gene Ontology (GO) pathway analysis and (**E**) STRING protein–protein interaction analysis of the DEGs upon d-Tyr administration in APP/PS1 mice.

Given that MCR activation elicits multiple beneficial effects in the hippocampus in APP/PS1 mice (e.g., restoring synaptic plasticity impairment, reducing Aβ level and deposition, and restoring glial cell homeostasis), we performed transcriptome analysis to examine the regulation of molecular phenotypes in d-Tyr–treated hippocampal slices from APP/PS1 mice. We showed that upon administering d-Tyr to hippocampal slices from APP/PS1 mice, 339 genes (i.e., 141 upregulated and 198 downregulated) were differentially expressed (i.e., log_2_ fold change ≥ 0.3 or ≤  − 0.3; *p* < 0.05) (Fig. 5A). Interestingly, the d-Tyr–induced transcriptomic changes in APP/PS1 mice were negatively correlated with the transcriptomic changes in APP/PS1 mice relative to the WT controls (*r* =  − 0.64, *p* < 0.0001; Fig. 5B). The expression levels of the differentially expressed genes in the d-Tyr–treated APP/PS1 mice resembled those in the untreated WT mice (Fig. 5C), suggesting that d-Tyr administration partially reverted the transcriptomic profile of APP/PS1 mice to that of the WT mice. Such restored expression was validated by quantitative RT-PCR for certain genes associated with microglial activation, including *Itgam* (*integrin subunit alpha M*, which encodes CD11b [cluster of differentiation molecule]) and *Aif1* (also known as Iba1) (Fig. 5, Supplemental Fig. 5). Accordingly, d-Tyr administration mitigated the marked upregulation of the transcript levels of these genes in the hippocampal slices from APP/PS1 mice—significantly so for *Itgam* (Supplemental Fig. 5A). Similarly, the transcriptional changes of the pro-inflammatory cytokine *Il6*, the astrocyte marker *Gfap*, *Chmp3* (*charged multivesicular body protein 3*), and *Zfp579* (*zinc finger protein 579*) were also validated (Supplemental Fig. 5C–F).

To examine the functional implications of restoring these transcriptomic signatures in APP/PS1 mice upon d-Tyr administration, we performed pathway analysis to annotate the identified differentially expressed genes. Both GO and protein–protein interaction analyses showed that the d-Tyr–upregulated genes were associated with homeostatic processes and responses to heat, unfolded protein, TGF-β (transforming growth factor-beta), topologically incorrect proteins, and stress; meanwhile, the d-Tyr-downregulated genes were associated with the regulation of metabolic processes, protein modification processes, macromolecule localization, histone modification, rRNA processing, cellular respiration, and microglial activation (Fig. 5D,E). Hence, the diverse molecular events caused by enhanced melanocortin signaling might provide insights into the molecular mechanisms underlying the observed shifts in glial reactivity and restoration of hippocampal homeostasis.

## Discussion

In this study, we demonstrated that the activation of melanocortin signaling ameliorates Aβ deposition and glial activation in an AD transgenic mouse model. We also showed that administration of the MCR agonist, d-Tyr, reduces amyloid burden in both the hippocampus and cortex, with a concurrent decrease in global neuroinflammation and activation of astrocytes and microglia. Specifically, MCR activation reduces neurotoxic astrocytes and microglial activation in a brain region-specific manner. Together with our transcriptome analysis revealing the downregulated expression of genes involved in microglial activation in d-Tyr–treated hippocampal slices, our findings suggest that microglia are one of the major cell targets that mediate how melanocortin/MCR signaling alleviates the pathological features of AD.

Considering that current therapeutic approaches for AD merely provide symptomatic relief, there is an urgent need for new curative treatments targeting disease modification^46^. The restoration of hippocampal synaptic plasticity, which forms the basis of learning and memory^47^, is a possible target for therapeutic intervention. Meanwhile, replenishment of POMC/MC4R signaling has been demonstrated to rescue synaptic plasticity impairment^21,32^. In addition to neuronal functions, we showed in this study that melanocortin/MCR replenishment alleviates the activation of microglia and astrocytes, thereby restoring cellular homeostasis, rendering MCRs and their agonists attractive targets for AD therapeutic development. As such, further work that expands our understanding of the cellular and molecular basis of α-MSH/MCR signaling in the hippocampus will be invaluable for exploring their therapeutic potential as a treatment paradigm for AD.

Glial cells play a pivotal role in the elicitation of chronic neuroinflammation and aggravation of AD pathology^8,9,38,39,48,49^. In the present study, MCR stimulation blunts the activation of astrocytes, specifically, a newly identified astrocyte subtype, the neurotoxic A1 astrocytes^11,50^. A1 astrocytes are described to mediate synaptic loss and impairment as well as initiating neuronal cell death, whereas loss of C3-expressing astrocyte signaling alleviates synaptic failure and AD pathology^51,52^. Thus, it is interesting to examine whether the beneficial effect of d-Tyr in APP/PS1 mice on multiple functions is mediated through the reduction in neurotoxic A1 astrocytes.

The prominent response of astrocytes to MCR activation in APP/PS1 mice in the present study warrants the examination of whether d-Tyr directly acts on astrocytes to deactivate them or, alternatively, functions via the modulation of other cell types. Indeed, activated microglia are known to release several inflammatory cytokines, including IL-1α (interleukin 1 alpha), TNF (tumor necrosis factor), and C1q (complement component 1, subcomponent q), which together mediate A1 neurotoxic astrocyte induction^11,12^. Meanwhile, our results show that MCR activation reduces the activation and proliferation of microglia. Accordingly, transcriptome analysis revealed that direct stimulation of MCRs in acute hippocampal slices from APP/PS1 mice restored the expression of genes associated with microglial activation, i.e. *Itgam* and *Aif1*, to their respective levels in WT mice. Both cell-surface CD11b and intracellular Iba1 are widely regarded as microglial markers. Microglial CD11b integrin is reported to play a role in neuroimmunity in the hippocampus^53^, while Iba1 is involved in membrane ruffling and phagocytosis in activated microglia^54^. Their downregulation on the transcript level in the hippocampus of APP/PS1 mice by d-Tyr treatment suggests that MCRs regulate the genes associated with microglial activation, including those involved in neuroimmunity and phagocytosis. Interestingly, Iba1 is reported to induce IL-6 secretion in mononuclear cells^55^, while IL-6 is reported to increase CD11b expression in the mouse brain^56^. The simultaneous alteration of their transcript expression patterns demonstrated in the present work indicates that MCRs potentially impact inflammatory and glial responses through IL-6–related plurifunctional regulation.

The role of melanocortin signaling in glial dynamics is further implicated in GO analysis, which revealed that d-Tyr treatment restores TGF-β response in acute hippocampal slices from APP/PS1 mice. TGF-β is a cytokine with potential neuroprotective effects mediated by glial cells^57,58^, while treatment of A1 astrocytes with TGF-β is suggested to decrease the transcript levels of the reactive astrocytes^11^. Interestingly, treatment in cultured rat astrocytes and microglia with a synthetic α-MSH analog was seen to stimulate the release of TGF-β as well as another anti-inflammatory cytokine IL-10^59,60^, which are hence possible mediators of melanocortin actions. Therefore, it is worthwhile to determine whether α-MSH/MCR functions via the regulation of the production and release of cytokines from glial cells in AD.

Besides influencing glial activity and inflammation, MCR activation through d-Tyr administration might modulate other systems such as the endosomal–lysosomal pathway. Quantitative PCR assessment of a few of the significantly differentially expressed transcripts from the RNA-Seq data revealed that d-Tyr-induced MCR activation restored the diminished level of *Chmp3* and *Zfp579* in the hippocampal slices from APP/PS1 mice to their respective levels in WT mice (Supplemental Fig. 5). While little is known about *Zfp579* other than its potential involvement in transcriptional regulation, CHMP3 is a well-documented core component of ESCRT III (endosomal sorting complex required for transport III), which sorts transmembrane proteins into lysosomes via the multivesicular body pathway^61^. Given that multivesicular bodies are involved in the localization of APP and amyloidogenic processing^62^, MCR activation might influence Aβ accumulation via CHMP3-associated APP trafficking.

To further explore the therapeutic potentials of the MCR system in AD, it is crucial to characterize the spatiotemporal expression profile of MCRs in AD progression. Among different MCRs, while MC3R is predominantly expressed in the hypothalamus, MC4R is widely distributed throughout the brain, with prominent expression across the hypothalamus and hippocampus formation^27–29^. In addition to neurons, MC4R expression is identified in microglia and astrocytes^59,63,64^. Cell-type-specific knockout of MCR(s) in AD mouse models may facilitate the dissection of the cellular mechanisms underlying the beneficial action of the melanocortin system. In sum, activation of the melanocortin system exerts beneficial effect on multiple functional aspects in AD condition and offers a potential target for therapeutic interventions in AD.

## Supplementary Information

Supplementary Information

## Abbreviations

A.U.: Arbitrary unit
AAV: Adeno-associated virus
AD: Alzheimer’s disease
Aif1: Allograft inflammatory factor 1
APP/PS1: APPswe, PSEN1dE9
Aβ: Amyloid-beta
C3: Complement component 3
CA: Cornu ammonis
CD11b: Cluster of differentiation molecule 11b
DEG: Differentially expressed gene
D-Tyr: [D-Tyr^4^]-melanotan II
GFAP: Glial fibrillary acidic protein
GO: Gene Ontology
GPCR: G protein-coupled receptor
Iba1: Ionized calcium-binding adapter molecule 1
Icam1: Intercellular adhesion molecule 1
Il1β: Interleukin 1 beta
Il6: Interleukin 6
Itgam: Integrin subunit alpha M
MCR: Melanocortin receptor
PBS: Phosphate-buffered saline
PBST: Phosphate-buffered saline-Triton X-100
POMC: Pro-opiomelanocortin
STRING: Search tool for the retrieval of interacting gene
TGF-β: Transforming growth factor-beta
Veh: Vehicle
WT: Wild-type
α-MSH: Alpha-melanocyte–stimulating hormone
β-actin: Beta-actin
