# Supplementary material for: Melanocortin receptor activation alleviates amyloid pathology and glial reactivity in an Alzheimer’s disease transgenic mouse model
Source: Sci Rep. 2021 Feb 23;11:4359. doi: 10.1038/s41598-021-83932-4 (PMC7902646; doi:10.1038/s41598-021-83932-4)
Supplement: Supplementary file 1 — Supplementary Information [file 41598_2021_83932_MOESM1_ESM.pdf]

SUPPLEMENTAL INFORMATION

Melanocortin receptor activation alleviates amyloid pathology and glial reactivity in an Alzheimer's disease transgenic mouse model

Jackie K.Y. Lau[1,2†], Min Tian[1,†], Yang Shen[1], Shun Fat Lau[1,2], Wing-Yu Fu[1,2], Amy K.Y. Fu[1,23], and Nancy Y. Ip[1,2,3]*

[1] Division of Life Science, State Key Laboratory of Molecular Neuroscience and Molecular Neuroscience Center, The Hong Kong University of Science and Technology, Clear Water Bay, Hong Kong, China

[2] Hong Kong Center for Neurodegenerative Diseases, Hong Kong, China

[3] Guangdong Provincial Key Laboratory of Brain Science, Disease and Drug Development, HKUST Shenzhen Research Institute; Shenzhen-Hong Kong Institute of Brain Science, Shenzhen, Guangdong 518057, China

[†] These authors contributed equally to the manuscript

[*] Correspondence should be addressed to:

Prof. Nancy Y. Ip

Division of Life Science, State Key Laboratory of Molecular Neuroscience and Molecular Neuroscience Center

The Hong Kong University of Science and Technology

Clear Water Bay, Hong Kong, China

Phone: +852-2358-6161

Fax: +852-2358-1552

E-mail: boip@ust.hk

Supplemental Figure 1

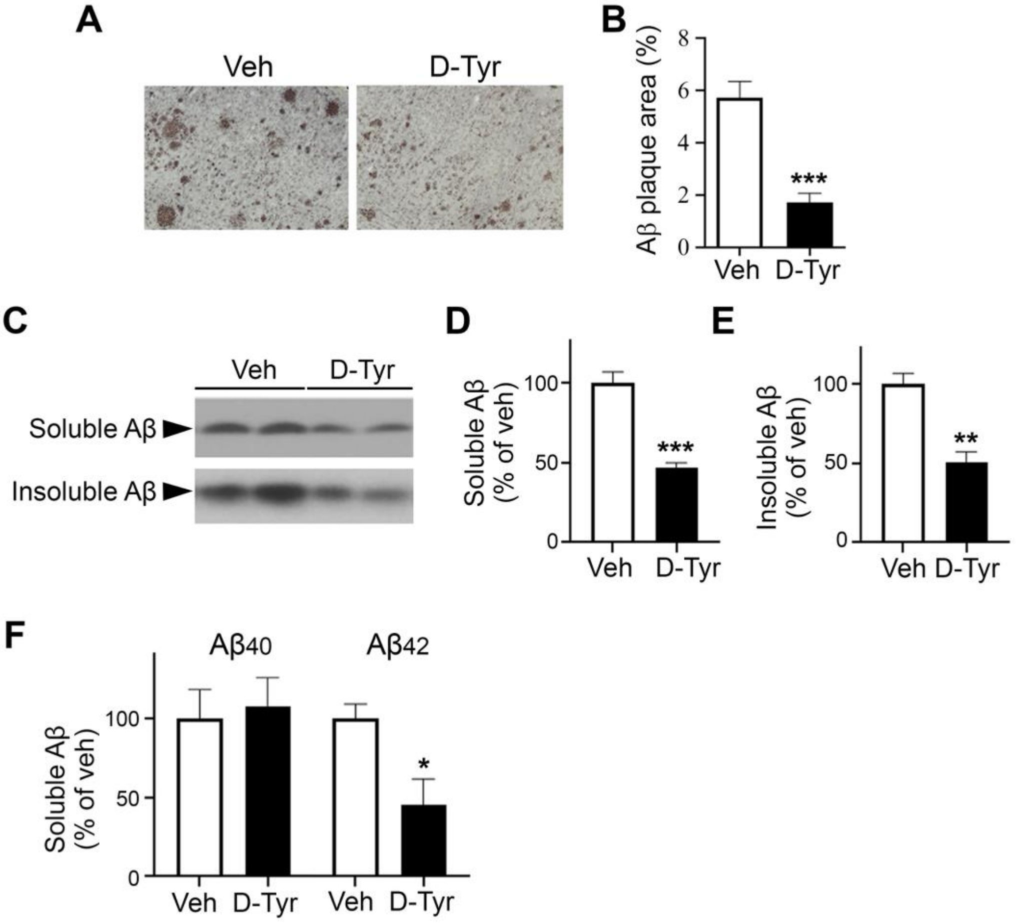

Supplemental Figure 1. Activation of melanocortin signaling ameliorates the amyloid pathology in the cortex in APP/PS1 mice. (A–B) Chronic D-Tyr administration reduces amyloid plaque deposition in APP/PS1 mice. Representative bright-field photomicrographs (A) and quantification (B) of 6E10-stained amyloid plaques in the neocortex on coronal brain sections of APP/PS1 mice following treatment with chronic D-Tyr versus control (Veh). Scale bar = 1 mm (*n* = 8–9 mice per group, 4 sections per mouse at 30-µm intervals). (C–E) Chronic D-Tyr administration reduces soluble and insoluble amyloid-beta (Aβ) contents. Representative western blot (C) and quantification of soluble (D) and insoluble (E) Aβ levels in cortical homogenates from APP/PS1 mice (*n* = 8–9 mice per group). Full-length blots are presented at the end of Supplemental Information. As the blots were cut prior to hybridization with antibodies, membrane edges were outlined with solid black lines. (F) Quantitative assessment (i.e., ELISA) of the relative levels of $A\beta_{x\text{–}40}$ and $A\beta_{x\text{–}42}$ isomers in the soluble fraction from the cortex of APP/PS1 mice (*n* = 4–5 mice per group). Data are the mean ± SEM of all mice from

each group examined (*$p < 0.05$, **$p < 0.01$, ***$p < 0.001$ for chronic D-Tyr vs. Veh treatment; Student's *t*-test).

Supplemental Figure 2

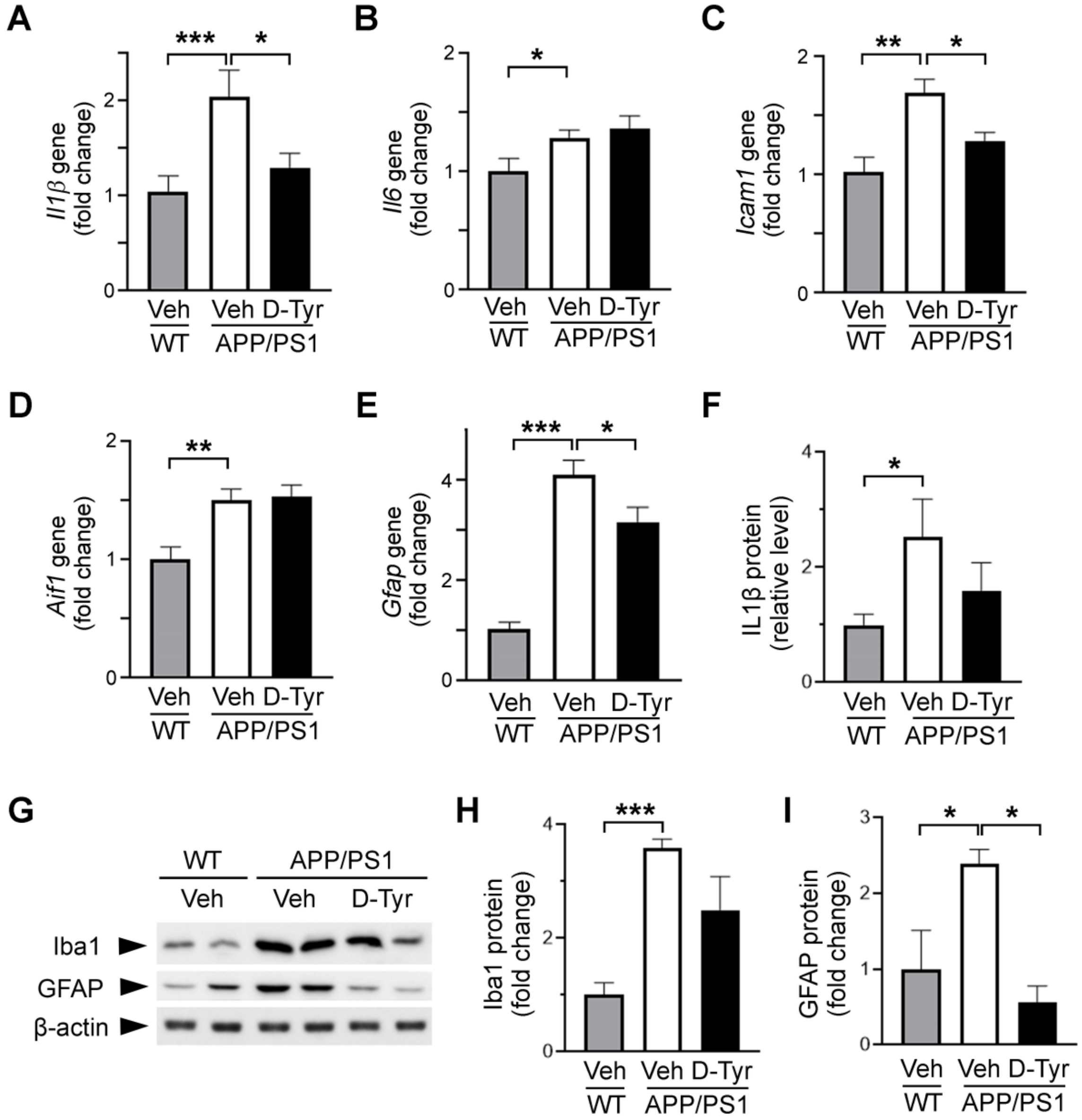

Supplemental Figure 2. Activation of melanocortin signaling modulates inflammatory responses in the cortex in APP/PS1 mice. Quantitative real-time PCR assessment of the levels of inflammatory genes in the cortex in wild-type (WT) mice treated with control (Veh) and APP/PS1 mice treated with chronic D-Tyr versus Veh. All measurements are normalized to the level of β-actin and presented as the fold expression relative to the average of the WT-Veh group. Transcript levels of *Il1β* (A), *Il6* (B), *Icam1* (C), *Aif1* (D), and *Gfap* (E). Quantitative assessment (i.e., ELISA) of the relative level of IL-1β (F) in the cortex. Representative western blot (G) and quantification of Iba1 (H) and GFAP (I) protein levels in cortical homogenates

from WT and APP/PS1 mice. Full-length blots are presented at the end of Supplemental Information. As the blots were cut prior to hybridization with antibodies, membrane edges were outlined with solid black lines. Data are the mean ± SEM of all mice from each group (n = 4–5 mice per group; *$p < 0.05$, **$p < 0.01$, ***$p < 0.001$; one-way ANOVA with the Bonferroni post hoc test).

Supplemental Figure 3

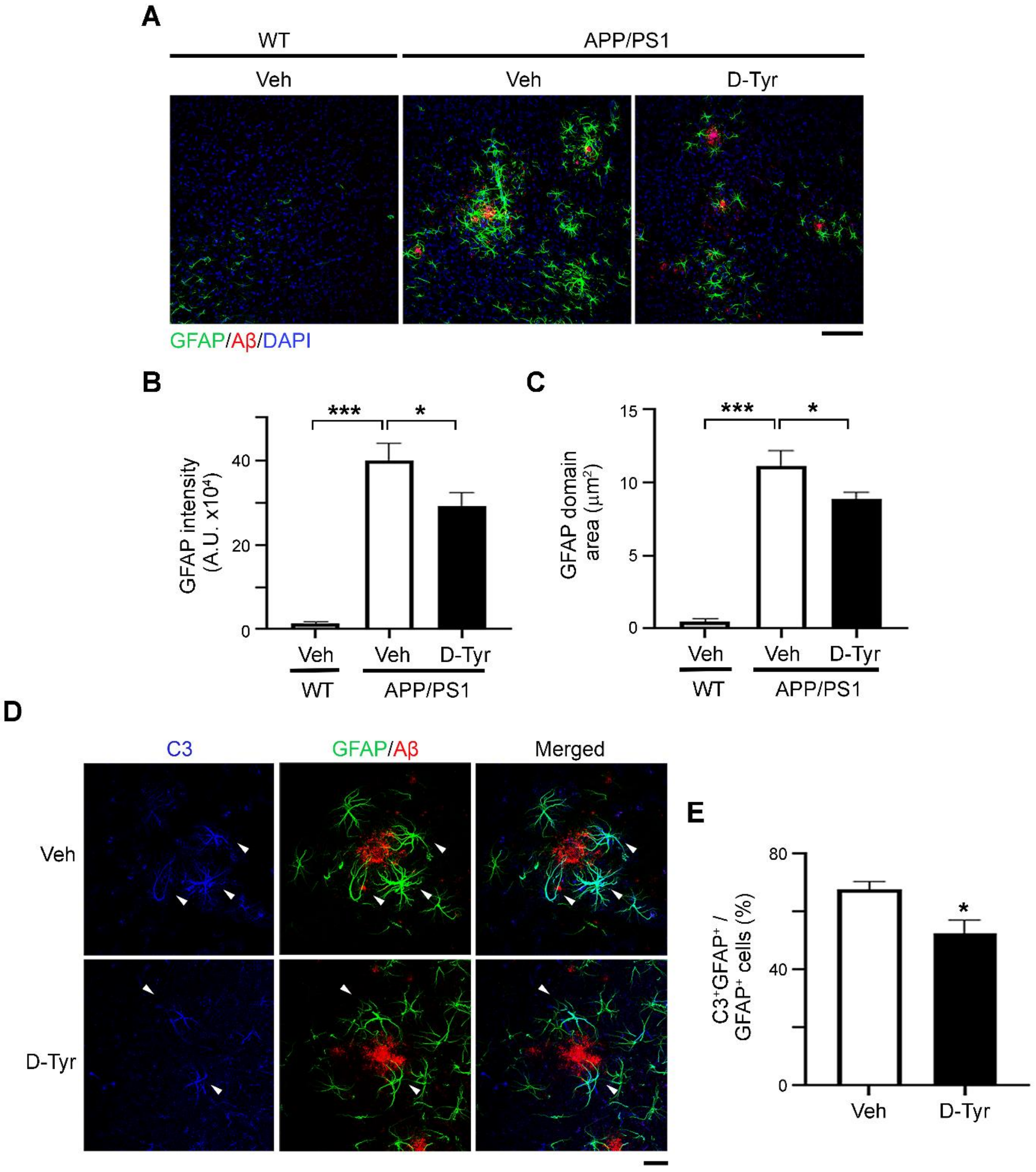

Supplemental Figure 3. Activation of melanocortin signaling reduces the reactivity of astrocytes in the cortex in APP/PS1 mice. (A–C) Chronic D-Tyr administration reduces GFAP expression in astrocytes in the cortex in APP/PS1 mice. Representative images (A), immunoreactivity in arbitrary units (A.U.) (B), and average domain areas (C) of GFAP in astrocytes in the cortical region on coronal brain sections of wild-type (WT) mice treated with control (Veh) and APP/PS1 mice treated with chronic D-Tyr versus Veh. Scale bar = 100 μm

(n = 9 mice per group; *p < 0.05, ***p < 0.001; one-way ANOVA with the Bonferroni post hoc test). (D, E) Chronic D-Tyr administration reduces the $C3^+$ (complement component 3) A1 subtype of reactive astrocytes. Representative images (D) and quantification (E) of $C3^+GFAP^+$ co-labeled astrocytes (white arrows) in the cortex in APP/PS1 mice treated with chronic D-Tyr versus Veh. Scale bar = 20 μm (n = 9 mice per group; *p < 0.05; Student's t-test).

Supplemental Figure 4

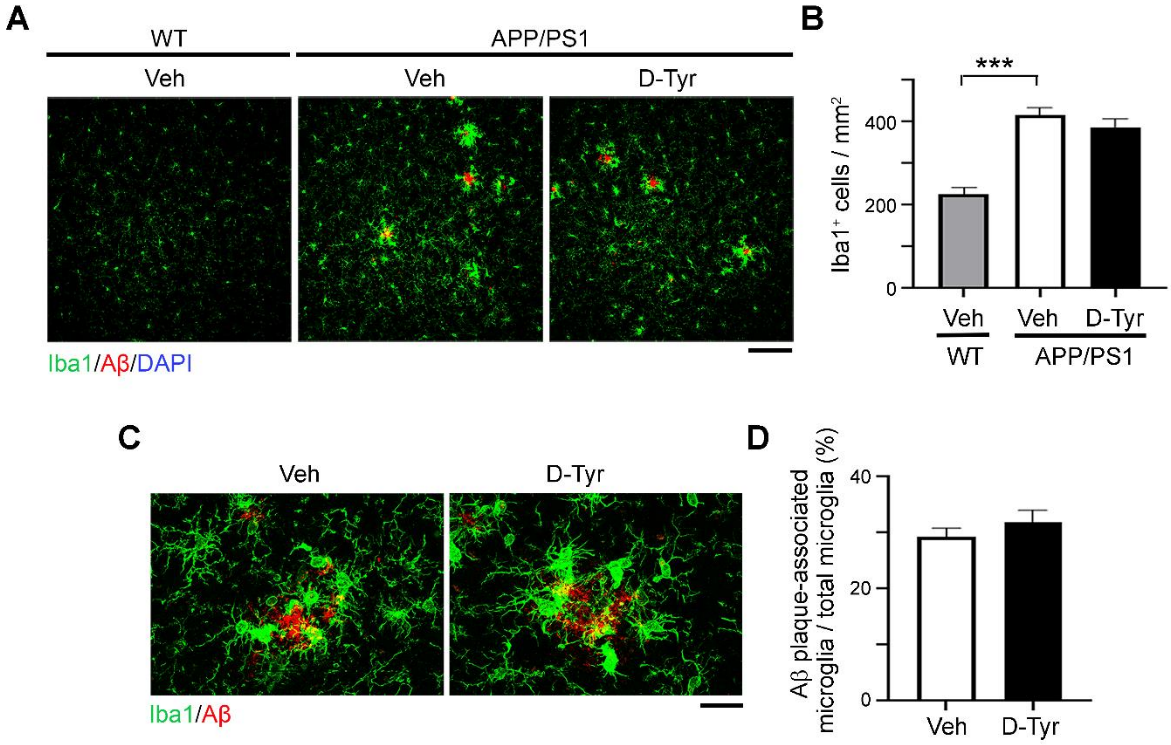

Supplemental Figure 4. Activation of melanocortin receptor signaling mediated by D-Tyr administration does not affect microglial reactivity in the cortex in APP/PS1 mice. Representative immunostaining (A) and quantification of the density (B) of microglia (labeled with Iba1) in the cortical region on coronal brain sections of wild-type (WT) mice treated with control (Veh) and APP/PS1 mice treated with chronic D-Tyr versus Veh. Scale bar = 100 μm (n = 9 mice per group; $***p < 0.001$ for WT vs. APP/PS1 mice receiving Veh treatment; one-way ANOVA with the Bonferroni post hoc test). (C, D) Chronic D-Tyr administration did not affect the association of microglia with amyloid plaques in the cortex in APP/PS1 mice. Representative images (C) and quantification (D) of amyloid plaque-associated Iba^+ microglia in the cortical region in APP/PS1 mice treated with chronic D-Tyr versus Veh. Scale bar = 20 μm (n = 9 mice per group; Student's t-test).

Supplemental Figure 5

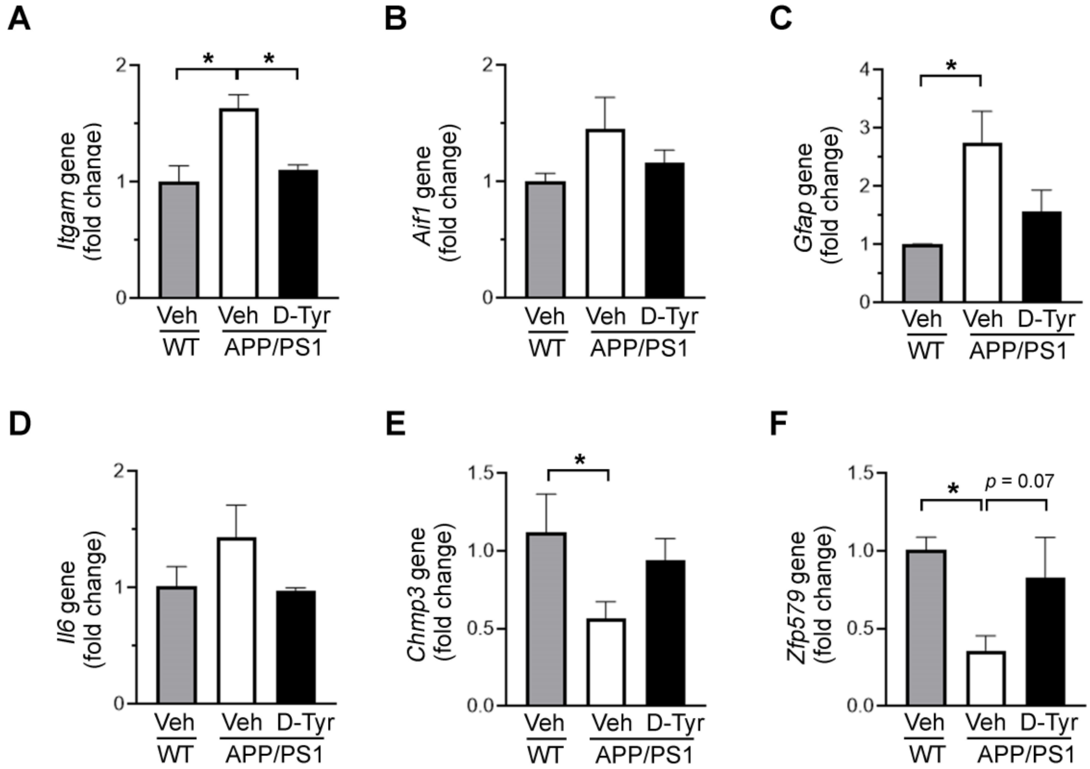

Supplemental Figure 5. Quantitative validation of differentially expressed transcripts in hippocampal slices from APP/PS1 mice. (A–F) Quantitative PCR showing the regulation of certain genes in acute hippocampal slices from control WT mice (Veh) and APP/PS1 mice treated with D-Tyr versus Veh. Transcript levels of *Itgam* (A), *Aif1* (B), *Gfap* (C), *Il6* (D), *Chmp3* (E), and *Zfp579* (F). All measurements are normalized to the level of β-actin and are presented as the fold expression relative to the average of the WT-Veh group. Data are the mean ± SEM of all mice from each group (n = 3–4 mice per group; *$p < 0.05$, **$p < 0.01$, ***$p < 0.001$; one-way ANOVA with the Bonferroni post hoc test).

Supplemental Information

Raw images of western blots

Figure 1E

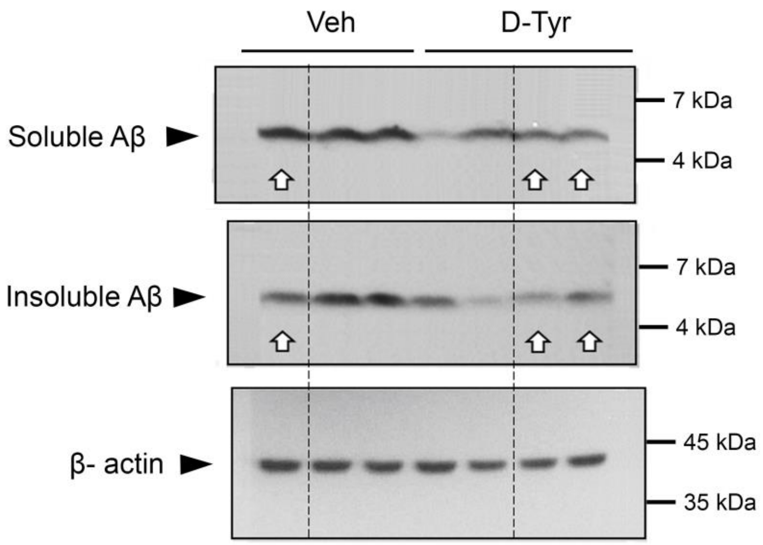

Supplemental Figure 1C

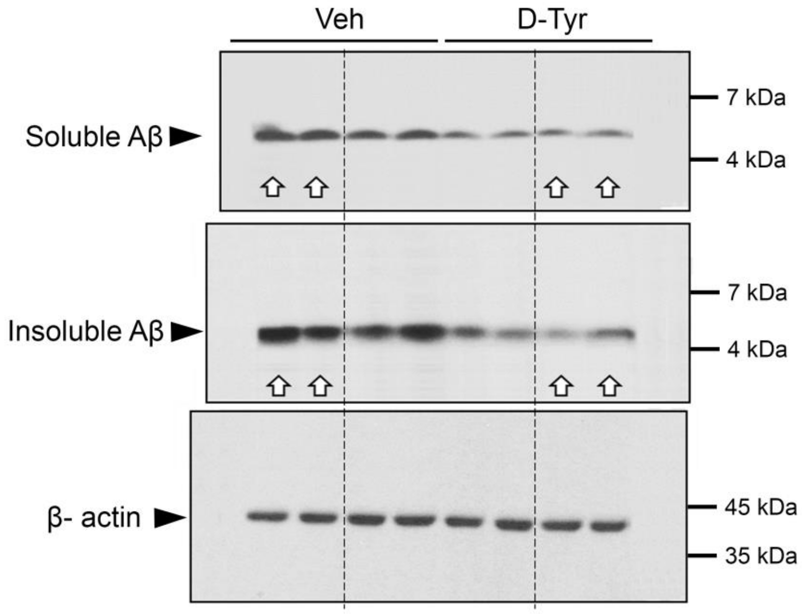

⇧ indicates band not included in main figures

Figure 2G

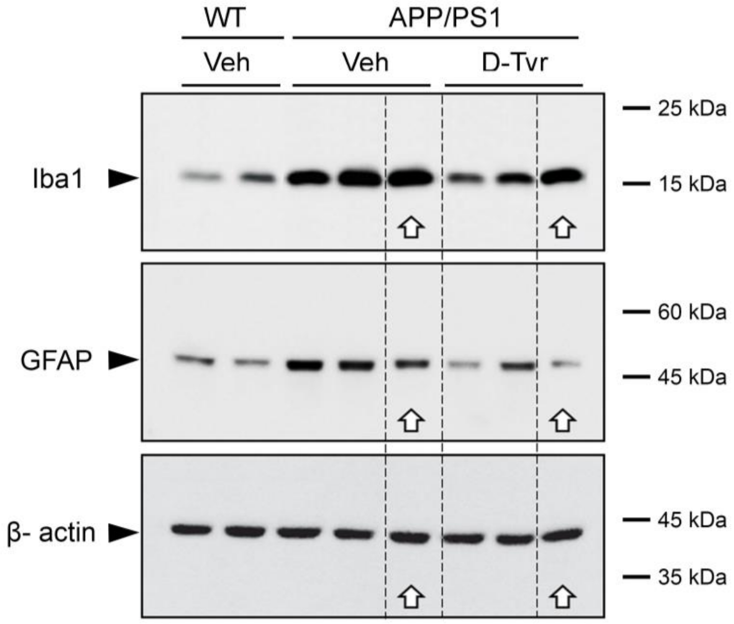

Supplemental Figure 2G

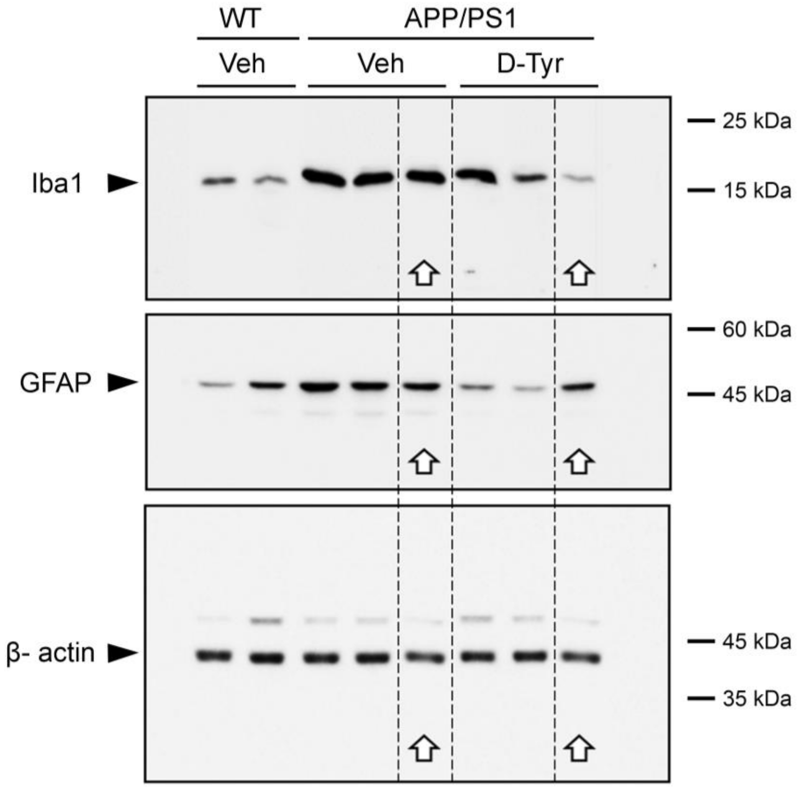

⇧ indicates band not included in main figures
